# Preliminary model-based validation of a biplane fluoroscopy system

**DOI:** 10.1186/1757-1146-7-S1-A53

**Published:** 2014-04-08

**Authors:** Joseph M Iaquinto, Richard Tsai, Quoc-Bao Vu, David R Haynor, Bruce J Sangeorzan, William R Ledoux

**Affiliations:** 1 RR&D Center of Excellence, VA Puget Sound Healthcare System, Seattle, WA, 98108, USA; 2 Depts. of Mechanical Eng, University of Washington, Seattle, WA, 98195, USA; 3 Radiology, University of Washington, Seattle, WA, 98195, USA; 4 Orthopaedics, University of Washington, Seattle, WA, 98195, USA

## Background

Biplane fluoroscopy can directly track the motion of bones and therefore measure joint kinematics. Our prior marker-based work has demonstrated the ability of our system to accurately and precisely track the motion of known objects (i.e., tantalum beads) [1]. In this study, we present the preliminary bone-based validation of our system by tracking the bones of the foot from cadaveric specimens.

## Methods

**Figure 1:**
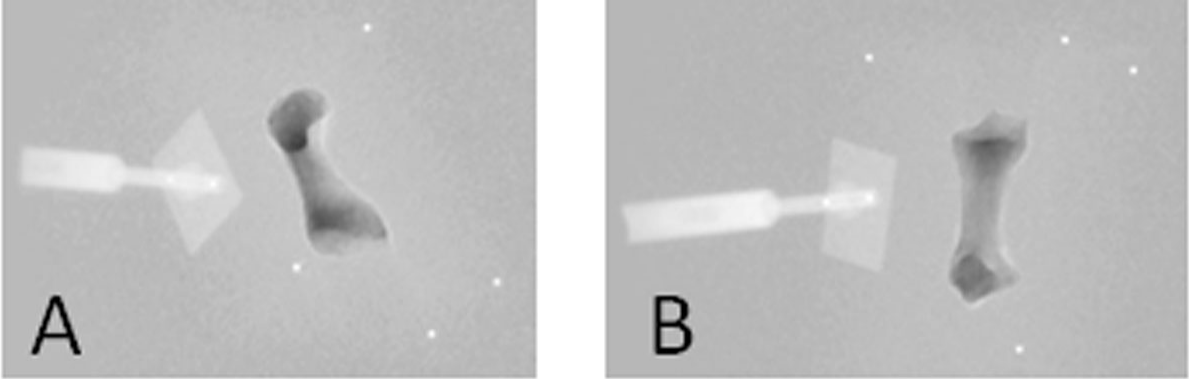
Fluoroscopy data with matched DRR overlay. Note: dense fluoroscope objects, such as the translational stage adaptor (to the left of the bone) show brighter than the background, while the DRR bone is displayed by pixel intensities darker than the background. Beads were not used.

Six bones (two each: calcaneus, talus and first metatarsal) were harvested from cadaveric feet and computed tomography (CT) scans of each bone were obtained. The CT data were used to create digitally reconstructed radiographs (DRRs) of each bone. Bones were attached to a single axis translational stage and imaged at 1000Hz at 13 discrete positions; data were averaged to an effective 100Hz sampling rate. DRRs of each bone were matched to X-ray data from each fluoroscope, and the three-dimensional position of each bone was calculated. Initial positions were manually found prior to the optimization algorithm calculating the “best” pose for every frame of each position (Figure 1). Accuracy is the root mean square (RMS) value of the difference between the software determined position and the known linear stage position. Precision is the standard deviation of the differences between these known and measured positions.

## Results

The translational accuracy for the entire data set (6 bones * 13 positions per bone * 10 frames per position = 780 frames) was 0.066mm with a precision of ± 0.062mm (Table 1).

**Table 1:** Individual bone accuracy and precision values, along with grand values for the six bone set.

	Calcaneus 1	Calcaneus 2	Talus 1	Talus 2	Metatarsal 1	Metatarsal 2	Grand
Accuracy	0.075	0.026	0.025	0.033	0.023	0.012	**0.066**
Precision	0.087	0.025	0.029	0.028	0.030	0.018	**0.062**

## Preliminary conclusions

These translational accuracy and precision values match well with other similar dual fluoroscopy systems studying areas of the body such as the spine [2] and knee [3]. Further, these values are an order of magnitude improvement over optical motion capture systems and have the ability to measure kinematics which are traditionally difficult to capture in the foot, such as talar motion. The full model-based validation of this system (which includes rotational and dynamic trials) is currently underway.
